# Cold-Shock Test Is a Practical Method for Selecting Boar Ejaculates Yielding Appropriate Seminal Plasma for Post-Thawing Supplementation

**DOI:** 10.3390/ani11030871

**Published:** 2021-03-18

**Authors:** Estíbaliz Lacalle, Andrea Núñez, Estela Fernández-Alegre, Itxaso Crespo-Félez, Juan Carlos Domínguez, Marta Elena Alonso, Raúl González-Urdiales, Felipe Martínez-Pastor

**Affiliations:** 1 Institute of Animal Health and Cattle Development (INDEGSAL), Universidad de León, 24071 León, Spain; elacf@unileon.es (E.L.); annugo95@gmail.com (A.N.); icresf00@estudiantes.unileon.es (I.C.-F.); jcdomt@unileon.es (J.C.D.); 2 Bianor Biotech SL, 24007 León, Spain; efernandez@bianorbiotech.es; 3 Topigs Norsvin España SLU (AIM Ibérica), 24225 León, Spain; rgonzalez@aimiberica.com; 4 Department of Animal Medicine, Surgery and Anatomy (Animal Medicine and Surgery), Universidad de León, 24071 León, Spain; 5 Department of Animal Production, Faculty of Veterinary, Universidad de León, 24071 León, Spain; marta.alonso@unileon.es; 6 Department of Molecular Biology (Cell Biology), Universidad de León, 24071 León, Spain

**Keywords:** boar, seminal plasma, cryopreserved semen, cold-shock test, data clustering, sperm motility, sperm physiology, chromatin status, osmotic resistance test, DNA fragmentation

## Abstract

**Simple Summary:**

The pig industry routinely uses artificial insemination with refrigerated semen. Cryopreserved semen has many advantages, but it is not widely used, partly because of unreliable results. The supplementation with seminal plasma (SP) could improve these results, but this fluid also presents variability. We evaluated if a simple cold-shock test (CST) could allow to easily identify the most suitable ejaculates for obtaining SP. Therefore, we tested 63 ejaculates, obtaining SP from the 4 showing higher quality (SPr, cold-shock-resistant) and lower quality (SPs, cold-shock-sensitive) after the CST. SPs and SPr pools supplemented thawed semen (20% SP) from six different boars, incubating at 37 °C and analyzing at times 0, 2, and 4 h. SPr was able to improve post-thawing sperm motility while maintaining viability. SPs had a similar but lower effect. SP in general seemed to stimulate sperm physiology, however decreasing membrane stability, acrosomal integrity, and disulfide bridges in the chromatin. This study supports the suitability of SP for improving thawed semen, with CST-selected ejaculates as preferable for this aim. Artificial insemination trials with thawed semen supplemented with SPr and SPs must validate the practical application of CST.

**Abstract:**

Artificial insemination (AI) with cryopreserved semen is still unreliable for extensive pig industry application. Adding seminal plasma (SP) could improve post-thawing quality, but its suitability could vary. We applied a simple cold-shock test (CST, 5 min at 0 °C) on neat semen for classifying ejaculates (*n* = 63) as resistant or sensitive, obtaining two SP pools (CST-resistant: SPr, sensitive: SPs). Subsequently, frozen/thawed spermatozoa from six boars were incubated (37 °C) in MR-A® extender (control), 20% SPr, or 20% SPs, and analyzed at 0, 2, and 4 h. SP improved total and progressive motility, with a higher effect for SPr and STR (*p* < 0.05), decreasing kinematic parameters VCL and VAP, ALH, and BCF. Sperm viability was unaffected. SP increased apoptotic and membrane disorder ratios, and acrosomal damage, not affecting the chromatin structure (DNA fragmentation and immaturity by SCSA), protamination (CMA3), or disulfide levels (mBBr). However, the proportion of spermatozoa with elevated free thiols (disulfide bridges reduction) significantly increased. Results support a stimulatory role of SP on thawed semen, with additional benefits from SPr. The effect of SP and especially SPr after AI should be tested since CST could be a practical test for selecting suitable ejaculates in AI centers.

## 1. Introduction

Artificial insemination (AI) with refrigerated semen is currently routine in the pig industry. The use of cryopreserved semen, widespread in the dairy industry, is still minimal [1] due to lower reproductive performance and higher costs [1,2]. However, the efficient application of cryopreserved semen in the porcine industry would be highly desirable since it would allow the long-term conservation of seminal doses, enhance the results of breeding programs, and contribute to better logistics and sanitary control [3,4].

The main reason for the poor performance of cryopreserved semen is cell damage during cooling, freezing, and thawing. This damage involves motility loss, plasma and acrosomal membrane changes, sperm chromatin damage, and decreased mitochondrial membrane potential [3,5,6]. Cold and osmotic shocks and oxidative damage induced by reactive oxygen species (ROS) are critical factors in these processes, and therefore there are many strategies aimed at reducing their impact [4,7]. Spermatozoa are vulnerable due to their physicochemical characteristics. The boar spermatozoon is especially susceptible to quick cooling (cold shock), and oxidative stress is due to the lipid composition of the plasma membrane, characterized by a low cholesterol content and a high proportion of unsaturated fatty acids [3,8]. Oxidative stress initiates lipid peroxidation in the membrane, subsequently affecting sperm functionality and, most critically, damaging chromatin integrity [9].

The susceptibility of spermatozoa to stress and damage depends on multiple factors. Thus, the diet, environment [10], and notably boar genetics play an essential role [6,11]. These factors express directly in sperm physiology and through changes in seminal plasma composition [12,13]. Seminal plasma (SP) is a fluid resulting from the secretions of the male accessory glands, which transports and protects spermatozoa at the time of ejaculation [14]. Its composition is very relevant for sperm physiology. Many of the proteins in SP coat the spermatozoa, preventing capacitation, modifying membrane composition, and modulating sperm metabolism and motility [15,16]. Besides, even though only a small amount of SP may enter the uterus (depending on the species), many studies have confirmed changes in the gene expression and immune response of the endometrium, including an improvement of spermatozoa function and embryo development [17,18,19,20].

Artificial reproductive techniques interfere with SP function by removing or diluting SP or, contrarily, lengthening SP and spermatozoa interaction [21,22]. In the pig industry, SP is typically removed or highly diluted as part of cryopreservation protocols [12]. Thus, the supplementation of thawed sperm with SP could be a strategy to improve its quality. SP produces an improvement in many sperm parameters, helping to maintain motility [5,12], reducing ROS production [23] and chromatin alterations [5], and overall preventing or reversing cryocapacitation [5,8,22,24]. Some studies report an improvement in the membrane and acrosomal integrity [25] and even fertility [26,27,28]. However, the significance of SP is variable among studies, and the effects on fertility are not entirely consistent (reviewed in Reference [27]). These contradictions may originate from the variable SP composition, differing between breeds, individuals, ejaculates, and even fractions within the same ejaculate [29,30]. Thus, some authors have proposed selecting boars for obtaining the most suitable seminal plasma considering the resistance of their spermatozoa to the cryopreservation (“good freezer” boars) [31].

Following these findings and aiming at improving the post-thawing results of boar spermatozoa, we have tested if the SP from boar ejaculates with different sperm resilience could act differently on thawed spermatozoa. Whereas the distinction between “good freezers” and “bad freezers” is adequate, more straightforward tests could be more practical. Since the boar spermatozoon is especially sensitive to sudden cooling, we devised a cold-shock test (CST) for testing ejaculates for spermatozoa resilience. Since the pig industry requires quick and cost-effective procedures, this could provide a practical test for the obtention of the most suitable SP for post-thawing supplementation.

## 2. Materials and Methods 

### 2.1. Reagents

General reagents and the fluorescent probes Hoechst 33342, propidium iodide, and peanut agglutinin (fluorescein isothiocyanate, FITC-conjugated) were purchased from Sigma-Aldrich (Merck KGaA, Darmstadt, Germany). MR-A^®^ extender for preparing the semen samples was produced by Kubus SA (Madrid, Spain). Consumables and solutions for flow cytometry were purchased from Beckman Coulter (Brea, CA, USA) and Thermo Fisher (Waltham, MA, USA). The fluorescent probes YO-PRO-1, merocyanine 540, and Mitotracker™ Deep Red were purchased from Thermo Fisher.

### 2.2. Experimental Design

The study was divided into two parts: (1) cold-shock resistance test and classification of boar semen samples according to their susceptibility to cold-shock resistance test and SP obtaining, and (2) testing the effect of the SP from resistant (SPr) or sensitive males (SPs) on the physiology of thawed spermatozoa. The general design is explained below, with procedural details being shown in specific sections.

#### 2.2.1. Experiment 1: Cold-Shock Resistance Test (CST), Boar Classification, and Seminal Plasma (SP) Preparation

Ejaculates from 63 boars (one per boar, only the rich fraction considered) were collected and extended at 10^9^ mL^−1^ in MR-A boar semen extender. The samples were transported and maintained at 17 °C. Five hours after collection, the samples were diluted to a final concentration of 50 × 10^6^ mL^−1^ in MR-A. Microtubes (1.5 mL) with 1 mL of extended samples were immersed in a water:ethanol (1:1) bath at 0 °C. After 5 min, they were removed from the bath and checked for motility, viability, and acrosomal integrity.

The data from the analysis was used for clustering the boars into four populations with a k-means algorithm. Five boars from each of the two most extreme populations were selected as resistant and sensitive. A new set of ejaculates from these boars was collected in the same manner, processed individually, and centrifuged twice in 15 mL tubes (800× *g*, 10 min at room temperature) to obtain SP. The top part of the supernatant was stored at −80 °C.

#### 2.2.2. Experiment 2: Effects of SPr and SPs on Thawed Boar Semen

The cryopreserved semen samples were stored in our cryobank (INDEGSAL, University of León) from previous studies [5,23], at 10^9^ mL^−1^. The doses used in this study were obtained from six Landrace boars and were selected for having at least 30% progressive motility after thawing. Two straws per male were thawed by immersion in a thermostatic water bath for 12 s at a temperature of 50 °C [32]. The contents of both straws were pooled and kept at 25 °C for 10 min.

SP thawing was performed on ice, centrifuging the tubes again and using the top supernatant. The SP were pooled in two groups according to the previous grouping (SPr and SPs), keeping them at 4 °C until use (<10 min) and warming up to 37 °C just before use.

Fifty-three microliters of thawed semen were extended in 1 mL of either MR-A (control, CTL) or MR-A with 20% SPr or SPs for a final concentration of 50 × 10^6^ mL^−1^. The samples were incubated at 37 °C and analyzed at 0 (no incubation), 2, and 4 h for sperm motility, membrane integrity (viability), acrosomal damage, apoptosis, mitochondrial activity, capacitation, and chromatin status. Sperm viability and acrosomal damage were analyzed again after carrying out an osmotic resistance test (ORT).

### 2.3. Semen Samples

Semen samples were donated by AIM Ibérica (Campo de Villavidel, León, Spain). Boars (Large White, Landrace, Duroc, and synthetic breeds) were kept following routine protocols for AI centers, were between 12 and 22 months old, and were subjected to semen collection weekly. They were kept under controlled conditions with a regulated temperature between 18 and 23 °C, and light/darkness hour cycles (12/12). AIM Ibérica provided semen doses from 63 boars (chosen randomly). The rich fraction of the ejaculate was collected and extended at 10^9^ mL^−1^ with MR-A. Ten-milliliter aliquots were transported refrigerated and kept until analyses at 17 °C in an IF450 incubator (Memmert GmbH, Schwabach, Germany) until the tests were carried out.

### 2.4. Assessment of Sperm Motility by CASA (Computer-Assisted Sperm Analysis)

Seminal samples were diluted to 25 × 10^6^ mL^−1^ in MR-A. Four microliters were loaded in a modified 20 µm Makler chamber (Sefi-Medical Instruments, Haifa, Israel) at 38 ℃. The CASA system consisted of a Nikon Eclipse 400 microscope (Nikon, Tokyo, Japan) equipped with a negative phase contrast system and a heating stage at 38 °C. Images at 10× were recorded with a Basler A312fc digital camera (Basler AG, Vision Technologies, Ahrensburg, Germany) into the CASA software ISAS^®^ v.1.2. (Integrated Semen Analysis System, PROiSER R + D, Paterna, Spain). At least three fields per sample (>300 spermatozoa) were acquired. The software configuration was set at 53 images/second, area range of 20–80 µm^2^, search radius of 11 µm, minimum images per sperm for ALH (amplitude of the lateral movement of the head) calculation of 7, the threshold for sperm motility of 10 µm/s, and progressive sperm as VAP > 25 µm/s (average path velocity) and STR > 45% (straightness). For each sample, the software provides the percentage of total motility (MOT, %) and progressive motility (PROG, %), as well as individual sperm parameters VCL (curvilinear velocity, µm/s), VAP (µm/s), VSL (straight-line velocity, µm/s), ALH (µm), BCF (beat-cross frequency, Hz), the derived linearity parameters (LIN) (linearity as VSL/VCL ratio, %), STR (VSL/VAP ratio, %), and WOB (wobble as VAP/VCL ratio, %), and the track-shape parameters Dance (DNC = VSL × ALH, µm^2^/s) and Dance mean (DNCm = DNC/VCL, µm).

### 2.5. Assessment of Sperm Physiology by Flow Cytometry

#### 2.5.1. Sperm Viability and Acrosomal Integrity

Peanut agglutinin (PNA, conjugated with the fluorescent fluorescein isothiocyanate (FITC) in this study) can bind to galactose residues on the inner side of the outer acrosomal membrane, allowing to identify spermatozoa with damaged acrosomes. Propidium iodide (PI) is a membrane-impermeant fluorochrome that binds DNA, enabling the identification of membrane-damage cells (dead) by labeling their nuclei. Samples were prepared at 2 × 10^6^ mL^−1^ in MR-A, adding 0.3 µg/mL PNA-FITC and 1.5 mM PI. After 15 min at 37 °C in the dark, the samples were analyzed employing flow cytometry. Hoechst 33342 (H342) was added at 4.5 µM for staining all nucleated events. Spermatozoa were classified into four subpopulations: viable with intact acrosome (PNA^−^/PI^−^), viable with acrosome damaged (PNA^+^/PI^−^), dead with acrosome intact (PNA^−^/PI^+^), and dead with acrosome damaged (PNA^+^/PI^+^). We used the proportion of acrosome-damaged spermatozoa (total PNA^+^) and the proportion of acrosome-damaged spermatozoa in the viable population (ratio of PNA^+^ considering only the PI^−^ viable population).

#### 2.5.2. Apoptosis-Like Features, Membrane Fluidity, and Mitochondrial Activity

We combined the fluorescence probes YO-PRO-1, merocyanine 540 (M540), PI, and Mitotracker™ deep red (MT) to evaluate apoptotic-like membrane changes, membrane fluidity (as a proxy for sperm capacitation or cryocapacitation), viability, and mitochondrial activity, respectively. Staining was carried out by diluting the sample to 2 × 10^6^ mL^−1^ in 300 µL of MR-A with 4.5 µM H342, 100 nM YO-PRO-1, 2 µM M540, 3 µM PI, and 100 nM MT. The tubes were incubated for 15 min at 37 °C in the dark. We used the proportion of YO-PRO-1^−^ spermatozoa as viable-nonapoptotic, YO-PRO-1^+^ within the PI^−^ population as the ratio of nonapoptotic within viable, M540^+^ within the YO-PRO-1^−^ population as the ratio of high-phospholipid-disorder plasma membrane within viable-nonapoptotic, and YO-PRO-1^−^/MT^+^ as viable-non apoptotic with active mitochondria.

#### 2.5.3. Osmotic Resistance Test

The osmotic resistance test (ORT) challenges the resistance of the plasma and acrosomal sperm membranes after stress induced by incubation under hypoosmotic conditions. Thus, resistance to osmotic stress is defined through two parameters: Ratio of viability and intact acrosomes [33]. In both cases, the ratio is obtained by dividing the value after osmotic treatment by this value under isosmotic conditions. Forty microliters of the sample were added to 400 µL of hypoosmotic MR-A (150 mOsm/kg) and incubated for 15 min at 37 °C. The samples were assessed for sperm viability and acrosomal integrity, as described in 4.5.1., and results were expressed as the change of the post-ORT values concerning the pre-ORT values (differences).

#### 2.5.4. Flow Cytometry Analysis of Sperm Physiology

The samples stained for assessing sperm physiology parameters (4.5.1–4.5.3) were run through a MACSQuant Analyzer 10 flow cytometer (Miltenyi Biotec, Bergisch Gladbach, Germany) equipped with three lasers: violet (405 nm), blue (488 nm), and red (635 nm). The photodetectors used to analyze the fluorescence from the different probes were: V1 with a 450/50 nm filter for H342, in the violet line, B1 with a 525/50 nm filter for PNA-FITC and YO-PRO-1, B2 with a 585/40 nm filter for M540, and B3 with a 655–730 nm filter for PI, in the blue line, and R1 with a 655–730 nm filter for MT, in the red line. The acquisition was controlled with the MACSQuantify™ software (Miltenyi Biotec, Bergisch Gladbach, Germany). The spermatozoa were identified with appropriate gates in FSC/SSC (forward/side scatter) and H342/SSC cytograms (H342^+^ events as spermatozoa), with a minimum of 10,000 events acquired per sample. Data, saved as FSC v.3 files, were analyzed by Weasel v. 3.4.2 (The Walter and Eliza Hall Institute of Medical Research, Victoria, Australia).

#### 2.5.5. Sperm Chromatin Assessment

Chromatin stability was assessed by SCSA (Sperm Chromatin Structure Assay) [9]. The technique is based on the denaturalization of sperm DNA and the shift of acridine orange fluorescence (AO, a DNA intercalating fluorochrome) from green (double-stranded DNA, dsDNA) to red (single-stranded DNA, ssDNA, resulting from fragmented DNA). Aliquots of samples from Experiment 2 were diluted in TNE buffer (0.01 M Tris-HCl, 0.15 M NaCl, and 1 mM Na_2_EDTA, pH 7.4) to 2 × 10^6^ mL^−1^. The analysis was carried out by mixing 200 µl of the sample with 0.4 mL of acid-detergent solution (0.08 M HCl, 0.15 M NaCl, and 0.1% Triton X-100, pH 1.2). After 30 s, the spermatozoa were stained with 1.2 mL of staining solution (6 μg/mL AO in 0.1 M citric acid, 0.2 M Na_2_HPO_4_, 1 mM Na_2_EDTA, and 0.15 M NaCl, pH 6.0). The tube was kept on ice for 3 min before flow cytometry analysis and run through a FACScalibur flow cytometer (Becton Dickinson) controlled by the acquisition software CellQuest v.3. We analyzed 5000 spermatozoa per sample, exciting the acridine orange with an Ar-ion 488 nm laser and using a 530/30 filter for the green fluorescence of dsDNA-bound AO and a 650 long-pass filter for the red fluorescence of ssDNA-bound AO. Data were saved in flow cytometry standard (FCS) v. 2 files, which were processed using the R statistical environment [34]. We calculated the DNA Fragmentation Index (DFI) for each spermatozoon as the red fluorescence to total fluorescence (red + green) ratio. From the DFI values, we obtained the percentage of spermatozoa with high fragmentation index (%DFI) as those with DFI > 250 and the percentage of spermatozoa with high DNA stainability (%HDS) defined as those events with green fluorescence above channel 650.

### 2.6. Data Analysis

Data analysis was performed in the R statistical environment v. 3.5.0 [34]. In Experiment 1, we used a non-hierarchical classification of the data (CASA and flow cytometry variables), using the k-means method (PAM algorithm, *cluster* library [35]), to classify the boars in groups according to the resistance of spermatozoa to the cold shock. Four groups were obtained as high, good, fair, and low, and visualized in the multidimensional space defined by the sperm quality variables by principal component analysis (prcomp in base R). The boars producing the ejaculates classified as excellent and poor were selected for the second experiment.

The results from Experiment 2 were analyzed by linear mixed-effects models, with the SP treatment (Control, SPr, SPs) and the incubation time (0, 2, 4 h) as fixed effects factors, and the boar (1–6) as the grouping factor in the random part of the model. The interaction treatment × incubation time was included, analyzing the factors as main effects when it was not significant or evaluating each factor within each other’s levels. When significant, the levels were compared by using multiple comparisons with Tukey’s method. Results are shown as mean ± CI95% (95% confidence interval) unless otherwise specified, and the significance threshold was set at *p* ≤ 0.05.

## 3. Results

### 3.1. Cold-Shock Resistance Test

**Figure 1:**
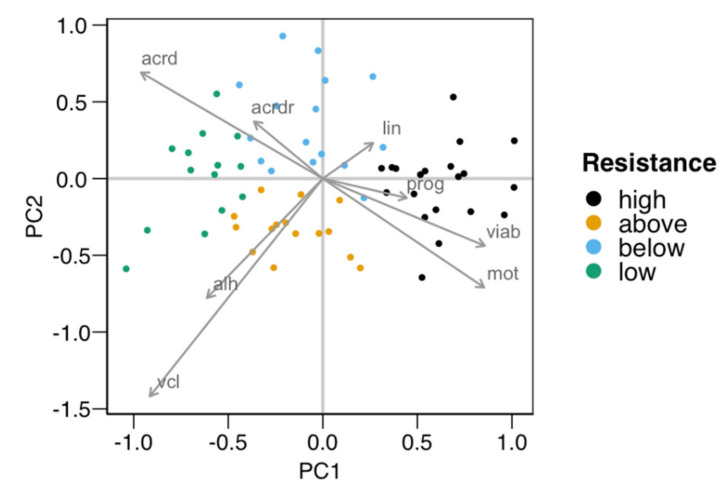
Principal components plot of the distribution of the males used in the first experiment for testing the resistance of their spermatozoa to the cold-shock test (*n* = 65). The four groups obtained with the k-means algorithm were distributed in pairs along the two axes defined by the two first principal components (PC1 and PC2, with 57% and 27% of the variance explained, respectively). PC1 separated groups “high” (higher viability and motility) from “low”, with PC2 separating “above” (higher velocity and motility) from “below”. The arrows show the loadings (a measure of association) of each variable relative to PC1 and PC2. The variables used in the clustering were total motility (mot), progressive motility (prog), curvilinear velocity (vlc), linearity (lin), the amplitude of the lateral movement of the head (ALH), viability (viab), total damaged acrosomes (acrd), and the proportion of viable spermatozoa with damaged acrosomes (acrdr). Five males from each “high” and “low” group were selected for producing the two pools of seminal plasma for the second experiment.

The cluster analysis classified the males based on their susceptibility to cold-shock, providing four groups. Table 1 shows the average values for the variables employed in the classification for each group of males. To better study the ejaculates’ distribution in the multidimensional space defined by these variables, we performed a principal component analysis (PCA). Figure 1 displays the graphical representation of the PCA for the first two principal components (84% explained variance), showing the observation assignment to each cluster, with the variable loadings for these principal components (PC1 and PC2) detailed in Table 2. As displayed by the variable loadings (arrows in Figure 1 and Table 2), sperm viability and motility were positively associated with PC1, with negative loadings for acrosomal damage (mainly total damage) and kinematic variables VCL and ALH. PC2 was negatively associated with most variables, but especially with VCL. Observations were mostly separated along PC1, with the “high” group with the highest PC1 values and the “low” one with the lowest ones. Intermediate groups “above” and “below” were separated along the PC2 dimension, mostly showing a difference in motility (higher total motility and velocity in “above”).

Five males from each of the “high” and “low” groups were selected for obtaining SP for the second experiment. As described in the Section 2, we prepared two pools termed SPr (seminal plasma from males providing spermatozoa with high resistance to the cold-shock) and SPs (seminal plasma from males providing spermatozoa with a low resistance to the cold-shock).

### 3.2. Testing the Effect of Seminal Plasma (SP) from Males with a High or Low Resistance to the Cold-Shock on Thawed Semen

#### 3.2.1. Sperm Motility

**Figure 2:**
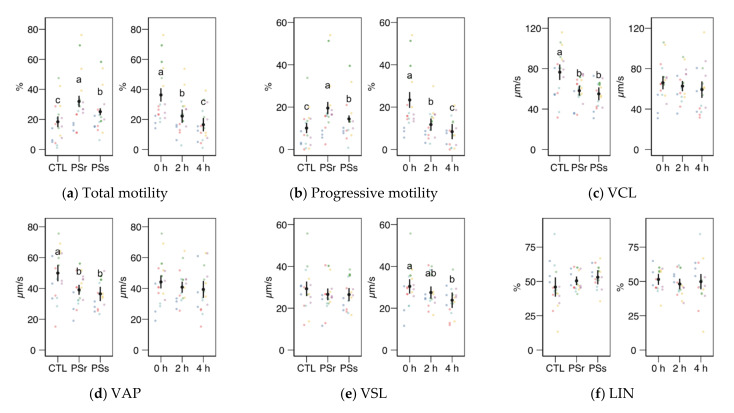

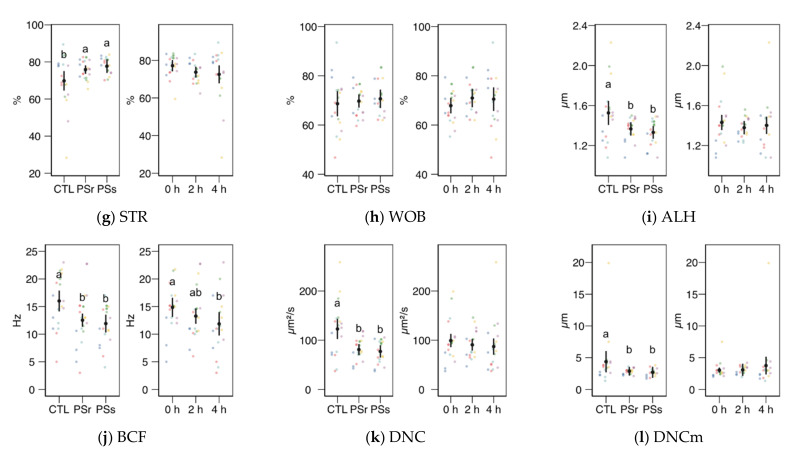
CASA (Computer Assisted Sperm Analysis) variables (mean ± CI95% with individual observations as dots, *n* = 6) during the incubation (4 h at 37 °C) of thawed boar spermatozoa with seminal plasma from boars whose spermatozoa were resistant or sensitive to the cold-shock (PSr and PSs) or incubated with just MR-A (control, CTL). The interaction treatment × incubation time was not significant, and therefore both factors were analyzed and plotted as main effects (therefore, the plotted observations correspond to the whole experiment, 6 × 3). (**a**) Total motility. (**b**) Progressive motility. (**c**) Curvilinear velocity. (**d**) Average-path velocity. (**e**) Straight-path velocity. (**f**) Linearity. (**g**) Straightness. (**h**) Wobble. (**i**) The amplitude of the lateral displacement of the sperm head. (**j**) Frequency of the flagellar beat. (**k**) Dance. (**l**) Dance mean. Since no interactions were significant, we show the results of the seminal plasma and incubation treatments separately as main effects. Different letters show significant differences among treatments or times (*p* < 0.05).

We observed no significant interactions between the treatment (control, SPr, and SPs) and the incubation time for the CASA variables, and therefore we studied the factors as main effects (displayed in Figure 2). In the case of total and progressive motility (Figure 2a,b), both the treatment and the incubation time showed significant effects (*p* < 0.001). Both SP increased the post-thawing motility above the control levels (incubated with the standard diluent MR-A), with SPr showing the highest results (*p* < 0.001 for total and progressive motility, SPr vs. CTL; *p* = 0.031 for total and *p* = 0.037 for progressive motility, SPr vs. SPs), on average doubling the proportion of motile and progressive spermatozoa compared to the control. The incubation time decreased total and progressive motility, irrespective of the supplementation. Whereas the straightness (STR) also increased in SPr and SPs sperm velocity as VCL and VAP (Figure 2c,d), and other motility parameters related to sperm hyperactivation such as ALH, BCF, DNC, and DNCm (Figure 2i–l), were higher in the Control samples (effect *p* < 0.001). The incubation time had no significant effect on these variables, except for inducing a significant decrease of BCF. The straight-line motility (VSL) significantly decreased with the incubation time, with no effect of the SP treatment. Among the linearity-related variables, STR increased with SP incubation (*p* = 0.008 for SPr vs. CTL, *p* = 0.062 for SPs), with no significant differences between PSr and PSs.

#### 3.2.2. Sperm Physiology

**Figure 3:**
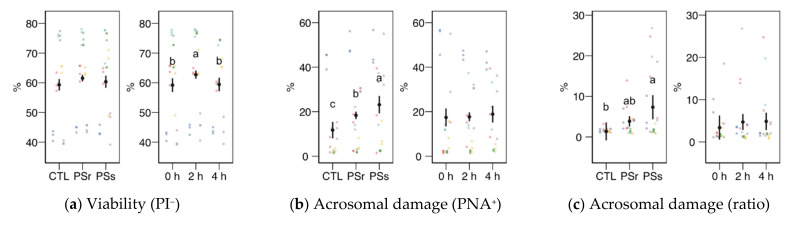

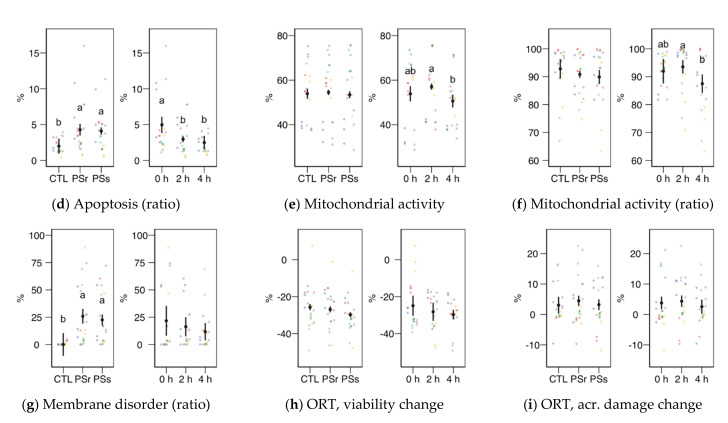
Sperm physiological variables obtained by flow cytometry (mean ± CI95% with individual observations as dots, *n* = 6) during the incubation (4 h at 37 °C) of thawed boar spermatozoa with seminal plasma from boars whose spermatozoa were resistant or sensitive to the cold-shock (PSr and PSs) or incubated with just MR-A (control, CTL). (**a**) Viable spermatozoa as PI^−^ events. (**b**) Acrosome-reacted (damaged, PNA^+^) spermatozoa. (**c**) Spermatozoa with damaged acrosome as the ratio of viable spermatozoa (PI^−^). (**d**) Apoptotic spermatozoa (YO-PRO-1^+^) as the ratio of viable (PI^−^). (**e**) Spermatozoa with active mitochondria (Mitotracker^+^). (**f**) Spermatozoa with active mitochondria as the ratio of viable spermatozoa (YO-PRO-1^−^). (**g**) Spermatozoa with high membrane disorder (M540+), as the ratio of viable spermatozoa (YO-PRO-1^−^). (**h**) Change of viable spermatozoa as PI^−^ after the osmotic resistance test (ORT). (**i**) Change of acrosome-reacted (damaged, PNA^+^) spermatozoa after ORT. Since no interactions were significant, we show the results of the seminal plasma and incubation treatments separately as main effects. Different letters show significant differences among treatments or times (*p* < 0.05).

Except for viability as YO-PRO-1^−^ spermatozoa, none of the physiology variables obtained by flow cytometry showed a significant interaction between the SP treatment and the incubation time; therefore, we analyzed each factor as the main effect (Figure 3).

**Figure 4:**
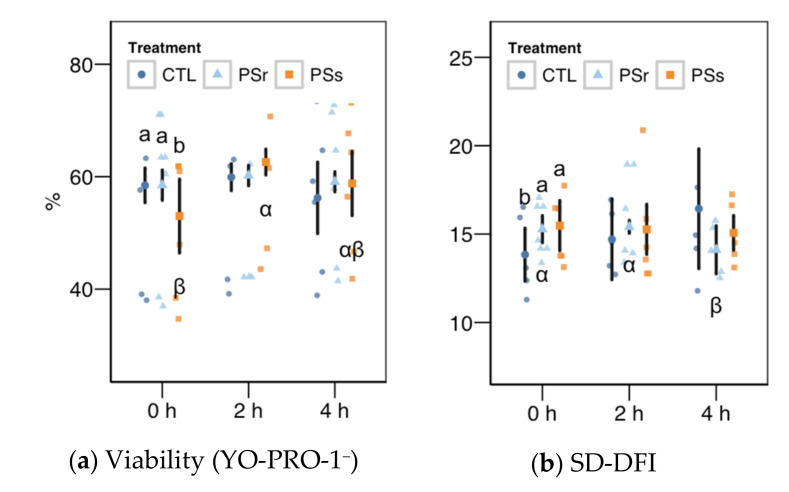
Results for the viability as YO-PRO-1^+^ events (**a**) and the SD-DFI (standar deviation of the DNA fragmentation index) parameter for SCSA (Sperm Chromatin Structure Assay) (**b**) as these variables showed a significant interaction between the treatment and the incubation (mean ± CI95% with individual observations as dots, *n* = 6) during the incubation (4 h at 37 °C) of thawed boar spermatozoa with seminal plasma from boars whose spermatozoa were resistant or sensitive to the cold-shock (PSr and PSs) or incubated with just MR-A (control, CTL). Different Latin letters show significant differences among treatments within each incubation time (*p* < 0.05), and different Greek letters show significant differences among incubation times within each treatment (*p* < 0.05).

Sperm viability as membrane integrity (PI^−^ events, Figure 3a) did not significantly vary among treatments, whereas it was significantly higher in the 2 h sampling. Considering viability as YO-PRO-1^−^ events (Figure 4a), we observed a significant interaction (*p* = 0.045) and then studied each factor within each level of the other. For this variable, SPs showed a significantly lower value at 0 h, with no significant differences at the other times. The values of SPs also showed a significant increase from 0 to 2 h, but not at 4 h. These variations in viability could be due to membrane permeability during the incubation instead of actual changes in sperm viability, as recorded previously for thawed spermatozoa [23]. The proportion of spermatozoa with active mitochondria, both overall (Figure 3e) and as the ratio of viable spermatozoa (Figure 3f), did not significantly differ among the treatments, being significantly lower at 4 h. 

Variables related to sperm capacitation, such as acrosomal damage or membrane disorder measured by merocyanine 540 (Figure 3b, c, g), increased when the thawed samples were incubated with SP. Total acrosomal damage also increased with SP, especially with SPs (*p* < 0.05). The incubation time did not significantly affect these variables. However, the apoptotic ratio (proportion of YO-PRO-1^−^ spermatozoa within the viable population, as PI^−^; Figure 3d) increased similarly in both SP treatments (*p* < 0.001) but significantly decreased from 0 to 2 h (*p* = 0.002) and 4 h (*p* < 0.001).

#### 3.2.3. Osmotic Resistance Test

Results for the osmotic resistance test (ORT) are shown in Figure 3h,i. The ORT effect on viability or acrosomal damage (change from pre-ORT as differences) was not significant, neither for SP supplementation nor incubation time.

#### 3.2.4. Sperm Chromatin Structure

**Figure 5:**
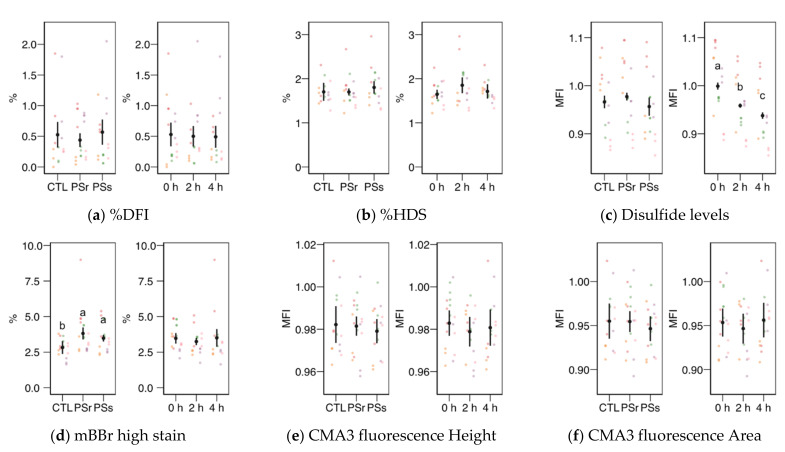
Sperm chromatin structure variables obtained by flow cytometry (mean ± CI95% with individual observations as dots, *n* = 6) during the incubation (4 h at 37 °C) of thawed boar spermatozoa with seminal plasma from boars whose spermatozoa were resistant or sensitive to the cold-shock (PSr and PSs) or incubated with just MR-A (control, CTL). (**a**) Proportion of spermatozoa with high DNA fragmentation index (DFI) from SCSA. (**b**) Proportion of spermatozoa with high DNA stainability from SCSA. (**c**) Disulfide bridge levels according to the monobromobimane (mBBr) stain. (**d**) Proportion of spermatozoa with high mBBr fluorescence levels. (**e**) Chromomycine A3 (CMA3) fluorescence levels according to the height of the signal. (**f**) CMA3 fluorescence levels according to the area of the signal. MFI: Median fluorescence intensity. Since no interactions were significant, we show the results of the seminal plasma and incubation treatments separately as main effects. Different letters show significant differences among treatments or times (*p* < 0.05).

Within the SCSA results, the SD-DFI analysis (the standard deviation of the DFI parameter as a measurement of spermatozoa heterogeneity) yielded a significant interaction between the treatment and the incubation time (Figure 4b). This variable showed values slightly but significantly higher at 0 h when samples were supplemented with SP (*p* = 0.041 for SPr and *p* = 0.021 for SPs), but not at other times. For SPr, values significantly decreased at 4 h (*p* = 0.018 vs. 0 h and *p* = 0.005 vs. 2 h). The other two SCSA variables considered in this study (Figure 5a, b), %DFI (DNA fragmentation) and %HDS (chromatin maturity), were affected neither by the supplementation nor the incubation time.

The levels of disulfide bridges (Figure 5c), a measurement of nuclear compaction, did not change among the PS treatments, with a decreasing trend with incubation time (*p* < 0.001 among times). The proportion of spermatozoa with the highest levels of monobromobimane fluorescence (Figure 5d) was higher for both SP concerning the control (*p* < 0.001 for SPr and *p* = 0.016 for SPs) with no change with incubation time. The chromomycin A3 fluorescence (related to histone levels) measured as height (Figure 5e) or area (Figure 5f) of the signal did not change significantly.

## 4. Discussion

This experiment demonstrated that SPr and SPs differ in their effects on the quality of the treated spermatozoa, following previous studies with seminal plasma from “good” and “bad” freezers [31]. In this case, the CST was a practical test for classifying the ejaculates. This methodology could be applied at AI centers, taking advantage of the routine removal of SP in many assisted reproduction protocols and enabling a good source for improving cryopreserved doses before their application.

There is evidence suggesting that SP enhances the post-thawing seminal quality [5,12,23,30,31,36] and can improve AI results [26,27,28]. Indeed, studies in boar [5] and ram [37] have indicated that the addition of SP enlarged sperm subpopulations with higher velocity and progressivity compared with the control. However, the effects of SP on thawed spermatozoa are diverse, even detrimental. This variability emerges from many factors (e.g., species or ejaculate fraction) that affect SP composition [38,39], therefore modifying sperm biology [31,40]. Comparative studies of the SP proteome have identified potential markers of seminal freezability in boars considered as “good” and “bad” freezers, such as fibronectin [41] or the activity of the enzyme N-acetyl-β-hexosaminidase [42].

In this context, the availability of a quick, cheap, and easy to perform test could enable the AI centers for discriminating a subset of males to obtain SP for ART. The cold-shock test is based on the observed variability in the resistance to cold-shock and cryopreservation of the spermatozoa from different males [4,43,44,45]. Even though the spermatozoa composition and the exposure to other fluids are relevant factors on their resilience and freezability [46,47], the SP composition could be very relevant [38,48,49]. Therefore, we first used a simple test to classify the potential donors according to the resistance of individual ejaculates to cold shock, testing in a second experiment if the SP from ejaculates defined as cold-shock-resistant (SPr) and cold-shock-sensitive (SPs) would differ in their ability for improving post-thawing sperm quality. Indeed, we obtained a large variability among ejaculates regarding the response to the cold-shock test.

Following previous experiments [23], the supplementation of thawed doses with SP (irrespective of the resistant/sensitive grouping) affected the plasma membrane characteristics (specifically to its permeability and fluidity, as shown in Figure 3 and Figure 4), increasing the proportion of reacted acrosomes too. That seems contradictory with reports showing that SP contains decapacitating factors [40,50,51]. However, SP could exert different effects in post-thawed spermatozoa. For instance, SP seemed to increase ROS during a post-thawing incubation in our previous study [23]. Since SP seems to benefit sperm motility and viability, we hypothesize that it might stimulate the metabolism of post-thawing spermatozoa, accelerating capacitation-like events in some sperm subpopulations (maybe the most affected by cryopreservation) while benefiting the survivability of others. Overall, this would result in a better performance of the thawed dose. This is compatible with a previous report showing a fertility improvement of cryopreserved doses treated with SP before AI [26]. Moreover, SP supplementation did not affect ORT resistance, indicating that any positive results of SP would be due to a modulation in the sperm physiology instead of a direct stabilization of the membranes. 

The use of SPr allowed for a more considerable improvement of total and progressive motility than SPs. SP improves the motility of post-thawed boar spermatozoa, according to previous studies [5,12,26], but our study shows a higher effect of ejaculates selected as resistant to cold-shock. A comparison of the responses elicited by SPr and SPs in motility and some flow cytometry parameters suggests that SPr could support the viability of a larger portion of spermatozoa, being more efficient in increasing their metabolic activity. Simultaneously, this increment did not reflect higher membrane instability or acrosomal damage (indeed, lower than SPs). SPs and SPr would similarly affect some pathways, whereas the effect on others would differ in intensity (reflecting in motility and viability). Future studies could test if these differences affect the formation of the oviductal reservoir [26,52] or fertility rates.

We studied the chromatin status by applying various techniques for assessing DNA fragmentation and structural features of the nucleus. This kind of analysis is of utmost importance since a relatively small increase in sperm DNA fragmentation (SDF) has been related to noticeable decreases in the average number of piglets [53,54]. In this study, SDF (estimated with the SCSA %DFI parameter) and the chromatin immaturity levels (%HDS) remained very low even for boar thresholds [53], affected neither by the cryopreservation nor by the incubation time. These results differ from a previous report, in which we could identify samples whose chromatin was sensitive to the post-thawing stress and could be partly ameliorated by adding SP [5]. Nevertheless, our results with the mBBr probe (disulfide levels) suggest some chromatin de-condensation during the incubation, with an effect of SP on the free sulfhydryl groups. The significance of these changes must be further evaluated, but they could be related to capacitation in a subset of spermatozoa, in line with the changes in membrane fluidity and acrosomal damage. These events might cause the reduction of disulfide bonds [55] and the concomitant increase of free thiol groups.

Considering the practical applications of this study, we must take into account that many AI companies and centers collect the entire ejaculate for preparing AI-doses, as this is more efficient [56]. This strategy might defeat the use of these ejaculates, as SP from post-SRF and sperm-free fractions could not be suitable for ART. However, the availability of a simple and quick test for selecting the males providing adequate SP could enable the occasional use of these boars for SRF-SP collection, improving the results of cryopreserved doses. Nevertheless, our study had two limitations in the first experiment since we studied one ejaculate per boar and obtained SP from the second set of untested ejaculates (however, shortly after testing the first set). We assumed that the seminal characteristics, including cold-shock sensitivity and SP composition, would be similar within each boar, as suggested previously [43]. However, this hypothesis must be tested. Although identifying boars providing suitable SP could be convenient, other studies have shown that many males could present a considerable between-ejaculate variability [57]. Molecular and cellular analyses could confirm if the SP maintains its properties as SPr and SPs throughout time and which components could vary among ejaculations and affect SP suitability.

Considering the second step of the ejaculate selection—the clustering analysis—we must point out that we used an unsupervised method (based on the k-means algorithms). However, each AI company could develop its sorting algorithm based on machine-learning methods using training data from their breeds. These supervised methods [58] would allow for a more specific and reproducible ejaculate selection for providing optimal SP.

Finally, we must point out that not only was SPs not detrimental for the thawed samples, but it also had a positive effect. We used pooled semen, and therefore the individual effects were not taken into account. Therefore, some of the cold-shock-sensitive samples could indeed contain SP of good quality. In this regard, the order of ejaculation influences sperm quality, and other reproductive fluids (e.g., epidydimal fluid) could influence “vanguard” spermatozoa from the first ejaculate fraction. Indeed, some authors have suggested that this first fraction (P1) might be the optimal one for ART because of this difference [46,47]. Therefore, when selecting ejaculates according to the resistance of their spermatozoa to cold-shock, there are many more factors involved than SP. Despite this limitation, the advantage of the cold-shock test over potentially more specific methods (e.g., proteomics) is the cost and the immediate implementation in the same AI center.

## 5. Conclusions

The cold-shock test could be a quick, cheap, and practical method for selecting suitable ejaculates for obtaining SP to improve the quality of thawed spermatozoa. When used at 20%, SP from cold-shock-resistant ejaculates (SPr) produced improvements in post-thawing sperm mobility. Additionally, the post-thawing supplementation of boar spermatozoa with 20% of SP, irrespective of its origin, produced changes in sperm physiology, including increased membrane instability and acrosomal damage, while maintaining sperm viability. Future studies should test differences in the SP proteome and metabolome [59] from resistant and sensitive ejaculates and if the application of SPr and SPs reflects on the fertility and prolificacy of the thawed samples.

## Figures and Tables

**Table 1:** Descriptive statistics (mean ± standard deviation (SD)) for the boar clustering according to the resistance of their spermatozoa to the cold-shock (*n* = 65).

Variable	High	Above	Below	Low
Number of boars in the cluster	20	15	16	14
Viability	63.1 ± 14.9	36.1 ± 9.8	28.8 ± 15	14.7 ± 7.6
Damaged acrosomes	23.5 ± 12.5	55 ± 18.7	66.1 ± 18.5	80.6 ± 9.9
Damaged acrosomes (ratio viable)	6 ± 3.2	7.5 ± 5.2	16.5 ± 15	23.9 ± 13.4
Total motility	61.4 ± 20	43.3 ± 14.5	18.5 ± 8.9	13.8 ± 9.5
Progressive motility	17 ± 7.7	8.3 ± 5	5 ± 2.8	2.3 ± 2
VCL	75.5 ± 17	132.6 ± 13.4	80.1 ± 18.9	131.8 ± 37.2
LIN	44.2 ± 10.7	30.8 ± 9.8	41.7 ± 13.3	27 ± 8.1
ALH	1.6 ± 0.3	2.7 ± 0.4	1.8 ± 0.3	2.9 ± 0.9

VCL: Curvilinear velocity; VAP: Average path velocity; VSL: Straight-line velocity; LIN: Linearity; STR: Straightness; WOB: Wobble; ALH: Amplitude of the lateral movement of the head; BCF: Beat-cross frequency.

**Table 2:** Variable loadings for the two first principal components (PC) plotted in Figure 1 (*n* = 65). The variance explained was 57% for PC1 and 27% for PC2.

Variable	PC1	PC2
Viability	0.428	−0.219
Damaged acrosomes	−0.481	0.346
Damaged acrosomes (ratio viable)	−0.182	0.186
Total motility	0.427	−0.355
Progressive motility	0.222	−0.063
VCL	−0.457	−0.71
LIN	0.133	0.116
ALH	−0.306	−0.389

VCL: Curvilinear velocity; VAP: Average path velocity; VSL: Straight-line velocity; LIN: Linearity; STR: Straightness; WOB: Wobble; ALH: Amplitude of the lateral movement of the head; BCF: Beat-cross frequency.

## Data Availability

The data presented in this study are openly available in Buleria (https://buleria.unileon.es (accessed on 22 February 2021)) at http://hdl.handle.net/10612/12975 (accessed on 22 February 2021).
